# Leveraging the Work Environment to Minimize the Negative Impact of Nurse Burnout on Patient Outcomes

**DOI:** 10.3390/ijerph18020610

**Published:** 2021-01-12

**Authors:** Amelia E. Schlak, Linda H. Aiken, Jesse Chittams, Lusine Poghosyan, Matthew McHugh

**Affiliations:** 1 Columbia University School of Nursing, New York, NY 10032, USA; lp2475@columbia.edu; 2 Center for Health Outcomes and Policy Research, Leonard Davis Institute of Health Economics, University of Pennsylvania School of Nursing, Philadelphia, PA 19104-4217, USA; laiken@nursing.upenn.edu (L.H.A.); mchughm@nursing.upenn.edu (M.M.); 3 Biostatistics Analysis Core (BECCA lab), Office of Nursing Research (ONR), University of Pennsylvania School of Nursing, Philadelphia, PA 19104-4217, USA; chittams@nursing.upenn.edu

**Keywords:** nurse burnout, burnout, work environment, Magnet, patient outcomes

## Abstract

Background: Burnout remains a persistent issue affecting nurses across the US health system. Limited evidence exists about the direct impact of nurse burnout on patient outcomes. This study explores the relationship between nurse burnout and mortality, failure to rescue, and length of stay, while also considering the effect of a good work environment. Methods: Cross sectional data from nurses and hospitals were used in conjunction with patient claims data. Multivariate logistic regression was used to study the relationship between nurse burnout, patient outcomes, the work environment, and Magnet status. Results: Higher odds of patient mortality, failure to rescue, and prolonged length of stay were found in hospitals that had, on average, higher nurse burnout scores. Good work environments were found to attenuate the relationship between nurse burnout and mortality, failure to rescue, and length of stay. Magnet status, another indicator of a good work environment, was found to attenuate the relationship between nurse burnout and mortality and failure to rescue. Conclusions: Improving the work environment remains a solution for hospitals looking to concurrently improve nurse burnout and patient outcomes. Administrators may look to the Magnet recognition program as a blueprint to better support nurses in providing safe, high quality care.

## 1. Introduction

Burnout has been studied extensively among nurses across the United States (US), and previous estimates have shown that between 35% and 45% of the US nursing workforce is burned out [1,2,3]. The World Health Organization (WHO) defines burnout as an occupational phenomenon, noting its inherent relationship to the workplace, and describes it as feeling emotionally exhausted, cynical, and ineffective in relation to one’s work, colleagues, and clients [4,5,6]. Unlike stress, the WHO recommends that burnout not be adapted for a personal context, but remain reserved as a phenomenon associated with work [4]. Burnout has been shown to be the consequence of a poorly designed work environment regardless of occupation, indicating that burnout is less about the type of work, but rather how the work is designed, distributed, and managed [7].

For nurses, having a supportive work environment means having the appropriate autonomy, adequate staff and resources, and good working relationships with physicians and management. When these conditions are in place, nurses have 28% lower odds of developing burnout [8]. Professional autonomy empowers nurses to exercise their discretion and respond efficiently to patient care issues. In fact, a study of dedicated AIDS units, characterized by high levels of nurse autonomy, found that nurses had, on average, a 5-point lower burnout score when compared with units that had a more physician-centric model [9]. In addition, a strong relationship between workload and burnout has been reported, with one study concluding that nurses were 78% more likely to report burnout in hospitals with poor staffing conditions [10], while another found that nurses had significantly lower levels of burnout in environments with ample staff and supportive services, reasonable workloads, and sufficient time to take 30 min breaks [11]. Along the same lines, nurses are sensitive to organizational factors affecting their working relationships with physicians and management. An organizational intervention increasing participative decision making among nurses reduced role ambiguity and role conflict [12]. Therefore, it is not surprising that in hospitals with high nurse participation in shared governance nurses had 36% lower odds of being burned out in comparison to hospitals with moderate levels of engagement in shared governance [13]. These trends have also been observed over time, providing greater evidence to support the consensus that poor work environments are a primary cause of nurse burnout [14]. Although one might think that nurse burnout results from the stress of working with seriously ill patients, the conceptualization of burnout as an organizationally produced phenomenon suggests it is more related to whether the working conditions are conducive to delivering patient care.

Research has also explored the effects of healthcare provider burnout on patient care. A 2016 systematic review found that nurse and physician burnout were associated with compromised patient safety, including higher rates of medical errors [15]. In addition to patient safety, burnout may jeopardize the rapport between nurses and their patients. Patients report lower satisfaction and are less likely to recommend their hospital when cared for in settings where there are higher numbers of burned-out nurses [2,16,17,18]. A contributing factor is that when nurses are burned out, they miss essential parts of patient care like medication delivery, communication, and surveillance [19]. Nurse burnout has also been linked with higher rates of workarounds, which are common in poor work environments, as nurses are forced to circumvent recommended policy for patient care due to an obstruction in their workflow [20,21]. While it is possible that nurses engage in workarounds or miss important aspects of patient care because they are burned out, an alternative explanation is that nurses and patients are vulnerable to the same aspects of a poorly designed workplace that lead to burnout.

Poor working conditions are not only predictive of nurse burnout, but also adverse consequences for patients. A 2019 meta-analysis by Lake and colleagues found that nurses in better work environments had 28% to 32% lower odds of developing job dissatisfaction, burnout, or intention to leave, and that patients had 8% lower odds of experiencing an adverse event or even death [8]. Similarly, Magnet hospitals, or hospitals recognized for excellence in nursing care, have been found to have analogous features of good work environments associated with lower levels of nurse burnout, improved job satisfaction, and lower intent to leave [22,23,24,25,26]. Magnet status has also been linked to improved patient outcomes. A 2013 study found that Magnet status was associated with 7.7% lower likelihood of patient mortality and 8.6% lower likelihood of failure to rescue (death after an in-hospital complication) [27]. While these studies established that features of good work environments are associated with lower burnout and improved patient outcomes, they have not considered the role of the work environment in mitigating the impact of nurse burnout on patient outcomes.

Two meta-analyses concluded that nurse and physician burnout are associated with lower levels of care quality, but highlighted a need for more quality clinical indicators, since much of the research to date has focused on indirect clinical indicators like clinician perceived quality [28,29]. Some studies linked nurse burnout to clinical outcomes such as mortality, adverse events, and length of stay; yet, these studies were limited in methodological approach and scope, and mixed in terms of results. For example, a study of 52 hospitals found nurse and physician burnout was not related to patient morbidity and mortality [30], but a subsequent study of 48 hospitals found that nurse and physician burnout was predictive of mortality but not of length of stay [31]. Importantly, however, both studies relied on convenience sampling of hospitals and nurses, limiting the generalizability of results. While one study of 48 hospitals revealed an unexpected relationship, that nurse burnout was associated with reduced length of stay [32], others have found a relationship between nurse burnout and hospital-acquired infections [33], and mixed results for adverse events [34,35]. For example, a 2019 study limited to one hospital reported that nurse burnout was associated with higher adverse events using a sample of 105 nurses and 150 patients [34], while another examined outcomes across three hospitals and found nurse burnout was associated with lower levels of adverse events [35]. These studies, while making important strides exploring nurse burnout and clinical patient outcomes, largely relied on smaller convenience samples of nurses and hospitals and did not adjust for important potential confounders, like the quality of the work environment. Our work in this study addresses this gap in the literature by assessing the impact of the work environment on nurse burnout’s relationship with patient outcomes.

In 1999, the National Academy of Medicine (formally the Institute of Medicine) published their report, “To Err is Human”, with the conclusion that health system design, rather than individual clinicians, was responsible for medical errors [36]. Similarly, the effect of nurse burnout on patient care and outcomes cannot be studied without examining the circumstances that enable it. Our study builds on prior research by examining the relationships between nurse burnout and clinical outcomes, including mortality, failure to rescue (death after experiencing an adverse event), and length of stay, while considering whether the nurse work environment could be leveraged to address nurse burnout and improve patient outcomes. We examined three hypotheses: (1) nurse burnout is associated with higher levels of patient mortality and failure to rescue as well as longer length of stay; (2) the nurse work environment attenuates the effect of nurse burnout on respective patient outcomes; (3) hospital Magnet status, as a proxy indicator for a good work environment, attenuates the effect of nurse burnout on respective patient outcomes.

## 2. Materials and Methods

### 2.1. Design and Data

This was a secondary analysis of four cross-sectional datasets linked and merged using a common hospital identifier. The 2015–2016 RN4CAST-US survey provided information on nurse burnout and hospital working conditions and randomly sampled and surveyed 30% of registered nurses from California, Pennsylvania, Florida, and New Jersey using a modified Dillman method [37]. There was a 26% initial response rate, and an intensive nonresponse survey was completed, yielding an 87% response rate [38]. Statistically significant differences were not observed between initial respondents and nonrespondents on the majority of hospital measures, minimizing the concern for nonresponse bias [38]. To obtain information on hospital features, publicly available data was gathered from the 2016 American Hospital Association (AHA) Annual Survey and the American Nurse Credentialing Center (ANCC). The AHA survey provided information on hospital structural characteristics, including the number of hospital beds, teaching status, technology status, and state, while the ANCC website provided information on hospital Magnet status. Patient data were obtained from state-based registries for California’s Office of Statewide Healthcare Planning and Development, Pennsylvania’s Health Care Cost Containment Council, Florida’s Agency for Health Care Administration, and New Jersey’s Department of Health and Senior Services. Patient data were used from the last quarter of 2014, all of 2015, and all of 2016 to be consistent with the timing of the RN4CAST survey. Both ICD-9 and ICD-10 codes were used, as the data spanned the transition in ICD coding.

### 2.2. Sample

The analytic sample included 20,496 nurses across 523 nonfederal, acute care hospitals from California, Pennsylvania, Florida, and New Jersey, of which 83 were Magnet hospitals. Consistent with previous empirical work, hospitals were included if they had at least 10 nurse respondents to aggregate individual reports of nurse burnout and the work environment to the hospital level [39,40]. In total, the patient sample included 1,939,878 surgical in-patients aged 18–99. We included patients that underwent vascular, orthopedic, or general surgery, as these groups include common procedures across hospitals and there are validated risk adjustment procedures [40,41,42,43].

### 2.3. Measures

Nurse Burnout was derived from the 9-item emotional exhaustion subscale of the Maslach Burnout Inventory, which was used in the RN4CAST-US survey. This portion of the survey asks respondents to report how often they experience feelings of emotional exhaustion in relation to their work [6]. There is strong conceptual and empirical justification for using the emotional exhaustion subscale, as it is identified as the core element of burnout [7,44,45,46], is the domain most frequently linked to outcomes [28], and has strong convergent validity [44,45,46]. Although the emotional exhaustion subscale provides a continuous measure of burnout, previous research [1,2,33] has largely relied on dichotomizing burnout so that nurses who score ≥27 on the subscale are considered highly burned out [6]. While dichotomizing burnout is helpful for identifying burnout, in terms of predicting outcomes, it is recommended to keep burnout in its continuous form so that differing degrees of emotional exhaustion can be linked to outcomes [6,47]. There are also noted weaknesses to dichotomization including information loss and diminished sensitivity in analyses [48]. Thus, we kept our measure of burnout in its continuous form and averaged it to the hospital level. This is consistent with the recommendations for use by the Maslach Burnout Inventory, which states that scores for a group of respondents can be aggregated [47,49]. We standardized the hospital level average to have a mean of zero and a variance of one so that a one standard deviation increase in the average nurse burnout score corresponds with the odds of each outcome.

The Nurse Work Environment was also derived from the RN4CAST-US survey, which asks nurses a series of questions from the Practice Environment Scale of the Nursing Work Index-Revised (PES-NWI) [50,51]. The PES-NWI is composed of 31 items contributing to 5 subscales: Nurse Participation in Organization Affairs; Nursing Foundations for Quality of Care; Nurse Manager Ability, Leadership, and Support of Nurses; Staffing and Resource Adequacy; and Collegial Nurse–Physician Relations [51]. The PES-NWI asks nurses to rate their level of agreement on a 1–4 Likert scale on whether certain features are present within their hospital. Cronbach’s α ranged from 0.84 to 0.93 and intraclass correlation was 0.84. We averaged individual nurse responses to the hospital level for each subscale and then averaged the subscales to create a continuous summary measure for each hospital [52,53]. We created categories of poor, mixed, and good by dividing the hospital level score into four equal quartiles. Consistent with previous research [14,18], a poor environment included the first quartile, a mixed environment was a combination of the 2nd and 3rd quartiles, and good environments included the 4th quartile.

Mortality was defined as in-hospital mortality within 30 days of the index hospital admission.

Failure to Rescue was defined as in-hospital mortality within 30 days of the index hospital admission after experiencing an adverse event, or an injury caused by medical treatment or management [54]. To create our measure of failure to rescue, we used a list of adverse events from the Agency for Healthcare Research and Quality’s Patient Safety Indicator-90. For this study, adverse events were defined as the development of at least one of the following conditions during the index hospitalization: pressure ulcer, iatrogenic pneumothorax, in-hospital fall with hip fracture, perioperative hemorrhage or hematoma, postoperative acute kidney injury requiring dialysis, postoperative respiratory failure, perioperative pulmonary embolism (PE) or deep vein thrombosis (DVT), postoperative sepsis rate, postoperative wound dehiscence, or unrecognized abdominopelvic accidental puncture/laceration [55,56].

Length of Stay was defined as the number of hospital days within 30 days of admission. Day 1 was considered the date of the index admission and the final day was the date the patient either died or was discharged. Patients who exceeded a 30-day length of stay were not considered, in alignment with quality guidelines, which recommend that outcomes with a standardized period of time be used to avoid bias in the results (i.e., 30 days from hospital admission) [57].

### 2.4. Covariates

We controlled for patient and hospital characteristics to isolate the relationship between nurse burnout and patient outcomes. For patient characteristics, we controlled for age, sex, race, transfer status, comorbidities, and surgical procedure. Included comorbidities were based on Elixhauser’s method of risk adjustment, which is validated for use in both ICD-9 and ICD-10 codes [58]. DRG codes were used for the identification of surgical procedure type (i.e., vascular, orthopedic, and general surgery).

We obtained hospital structural characteristics from the AHA data set and controlled for bed size, teaching status, technology status, and state. We categorized bed size into ≤100 beds, 101–250 beds, or >250 beds. Teaching status was classified into nonteaching with no residents/fellows, minor teaching with a ratio of 1:4 residents/fellows to bed, or major teaching with a ratio of >1:4 residents/fellows to bed. Technology status was dichotomized with high technology hospitals being identified as those with the capacity to perform open heart surgery and/or organ transplant. State included California, Pennsylvania, Florida, and New Jersey.

### 2.5. Data Analysis

To show the variation in nurse burnout across hospitals, chi-squared tests of significance for categorical variables and one-way analysis of variance (ANOVA) for continuous variables were used in Table 1. Frequencies and percentages were used to describe the nurse sample in Table 2 and the patient sample in Table 3. For statistical modelling, we used multivariate regression with adjustments for the clustering of patients within hospitals using Huber–White sandwich estimators [59,60]. Logistic regression was used to estimate binary outcomes, including mortality and failure to rescue, while zero truncated negative binomial regression was used to estimate length of stay. We sequentially tested our models, starting with the unadjusted model to estimate the relationship between nurse burnout and each of the patient outcomes. Model 2 stepped in patient characteristics and Model 3 stepped in hospital characteristics. We estimated the effects of the nurse work environment and Magnet status separately in Models 4 and 5 and then together in Model 6. All statistical analyses were completed in STATA Version 16.1 (StataCorp LLC, College Station, TX, USA) with the significance level set at <0.05. We completed a power analysis for mortality, our primary outcome of interest, in PASS 16 [61]. Based on our inclusion criteria, the analytic sample included 523 hospitals with a total of 1,939,878 surgical patients (about 3709 surgical patients per hospital). With this sample size, we were able to detect an odds ratio as low as 1.051 (80% power; α= 0.05; ρ= 0.100).

## 3. Results

### 3.1. Descriptive Characteristics of Hospitals

Table 1 presents the descriptive characteristics of the 523 hospitals. Hospitals were divided into quartiles based on the average nurse burnout score, with the 1st quartile including those hospitals with the lowest average burnout score (1st quartile mean = 16.6, range 8.3–18.6) and the 4th quartile including hospitals with the highest average burnout score (4th quartile mean = 25.7, range 23.2–31.9). The average nurse burnout score for the entire sample was 21 with a possible range of 0–54 (higher scores indicate higher levels of burnout) [5]. The average nurse burnout score varied significantly by work environment (*p* < 0.001). For example, among hospitals with good work environments, less than 2% were in the highest quartile of burnout compared with almost 60% which were in the lowest quartile of burnout. In contrast, over half of hospitals with poor work environments (54.2%) were in the highest quartile of burnout.

There were 83 Magnet hospitals in our analytic sample. We observed a similar relationship between Magnet status and the quartile of burnout as we did with work environment. We found that almost 40% of Magnet hospitals were in the lowest quartile of burnout (*p* < 0.001). Nurse burnout also varied significantly across other hospital features, including bed size (*p* = 0.021) and teaching status (*p* = 0.046).

### 3.2. Descriptive Characteristics of Nurses and Patients

Characteristics of the nurse sample are presented in Table 2 (*n* = 20,406). Characteristics of the patient sample are presented in Table 3 (*n* = 1,939,878). Our patient sample had almost a 1% mortality rate within 30 days of hospital admission, and just over 4% of patients that experienced an adverse event perished (i.e., failure to rescue). The average hospital length of stay was 4.3 days.

The relationship between nurse burnout and patient outcomes is presented in Table 4. We observed a significant relationship between nurse burnout and 30-day in-hospital mortality (OR = 1.06, *p* = 0.003), meaning that a one standard deviation increase in the average nurse burnout score was associated with a 6% increase in the odds of mortality. Even after controlling for patient and hospital characteristics (Model 3), this relationship remained significant (OR = 1.05, *p* = 0.023). By squaring the unrounded odds ratios, we observed that a two standard deviation increase in the average burnout score was associated with an 11% increase in the odds of mortality after accounting for patient and hospital characteristics (1.051535^2^ = 1.10572586). We also found significant relationships between nurse burnout and failure to rescue and length of stay even after adjusting for patient and hospital characteristics (Model 3). Specifically, a one standard deviation increase in the average nurse burnout score was associated with a 5% increase in the odds of failure to rescue (OR = 1.05, *p* = 0.037) and a 2% increase in the overall length of stay (IRR = 1.02, *p* = 0.013). A two standard deviation increase in average nurse burnout score was associated with a 10% increase in the odds of failure to rescue (1.046676^2^ =1.09553065) and a 3% increase in overall length of stay (1.016263^2^ = 1.03279049).

### 3.3. Effect of the Work Environment on the Association between Nurse Burnout and Patient Outcomes

In alignment with our second hypothesis, the work environment attenuated the effect of nurse burnout on mortality, failure to rescue, and length of stay, as indicated by a drop in the burnout odds ratio as well as a corresponding reduction in statistical significance. In addition to the attenuation of the association of nurse burnout with patient outcomes, we found that the work environment had a statistically significant effect on outcomes. A change in the work environment from poor to mixed or mixed to good was associated with a 14% drop in the odds of 30-day in-hospital mortality (OR = 0.86, *p* < 0.001). We had similar findings for failure to rescue and length of stay with a change in the work environment from poor to mixed or mixed to good being associated with a 12% drop in the odds of failure to rescue (OR = 0.88, *p* < 0.001) and a 4% drop in overall length of stay (IRR = 0.96, *p* = 0.003).

### 3.4. Effect of Magnet Status on the Association between Nurse Burnout and Patient Outcomes

We hypothesized that hospital Magnet status, like the work environment, would attenuate the effect of nurse burnout on patient outcomes. To assess this, we modelled the effects of the nurse work environment and Magnet status on outcomes independently in Models 4 and 5 and then together in Model 6. Focusing on hospital Magnet status in Model 5, we noted trends consistent with the work environment effect observed in Model 4. The presence of Magnet in the model for both 30-day in-hospital mortality and length of stay was associated with an attenuation in the burnout odds ratio and a loss of burnout’s statistical significance. Beyond that, Magnet status was also associated with a 18% drop in the odds of 30-day in-hospital mortality (OR = 0.82, *p* < 0.001) and a 13% drop in the odds of failure to rescue (OR = 0.87, *p* = 0.003). However, there was an exception for length of stay. The presence of hospital Magnet status in the length of stay model was insignificant (IRR = 0.99, *p* = 0.464), and there was no attenuation of burnout’s effect on length of stay (IRR = 1.01, *p* = 0.030).

In Model 7, the work environment and Magnet status were jointly modelled because although Magnet hospitals have better work environments on average, it does not perfectly account for all the variation in the work environment. Consistent with Models 4 and 5, we observed an attenuation and loss of significance for burnout’s effect for all three outcomes. Concurrent significance in the work environment and Magnet status was observed for 30-day in-hospital mortality and failure to rescue.

## 4. Discussion

Using a large sample of hospitals and patients, we assessed how widespread nurse burnout in US hospitals affects patient outcomes. We specifically showed that patients cared for in hospitals with high levels of nurse burnout have higher odds of patient mortality, failure to rescue, and longer length of stay, adding further evidence that there are life-threatening consequences for patients when nurses are burned out [31]. Our findings provide additional support for prior research evaluating the relationship of nurse burnout with nonclinical outcomes, such as nurse perceived quality and safety [15,28,29], and builds on it by situating our investigation of nurse burnout’s effect on patient outcomes within the context of the work environment. We demonstrated that the work environment lessens the impact of nurse burnout on mortality, failure to rescue, and length of stay. These findings suggest that a good nurse work environment has benefits beyond mitigating nurse burnout. Specifically, a good work environment could also be leveraged to minimize additional, avoidable sources of death among patients and reduce the length of hospitalization.

Much of the previous research exploring the effect of nurse burnout on patient outcomes has not used direct clinical outcomes, nor adequately considered the context of the broader work environment in creating situations ripe for both poor patient and negative nurse outcomes. Instead of focusing on how the experience of burnout affects personal productivity and quality, our study highlights another explanation: work quality associated with burnout is reflective of the work environment. Our analysis suggests that the work environment may account for not only nurse burnout, but also poor patient outcomes, partially because nurse burnout is low in hospitals with good work environments. In fact, we found that only 1.5% of nurses in good work environments are in the highest quartile of burnout.

To further explore the relationship of the work environment to burnout and outcomes, we considered hospital Magnet status, an external recognition that hospitals can achieve. Magnet hospitals are recognized for excellence in nursing care and have many of the features of good work environments, such as improved nurse autonomy, better staffing, and supportive management [62,63]. Our investigation revealed that Magnet status was associated with a reduction in the effect of nurse burnout on mortality and failure to rescue, but not with length of stay. Previous research has separately observed relationships between Magnet and lower levels of nurse burnout [22] as well as shorter length of stay [64,65]. Furthermore, some of the differences we observed between our work environment and Magnet findings were likely because Magnet and our measure of the work environment, while conceptually similar, were not perfect substitutes. This is also indicated by the concurrent statistical significance of both the work environment and Magnet in Model 7, suggesting that even after accounting for the work environment, Magnet status confers additional benefit in terms of these outcomes.

Twenty years ago, the National Academy of Medicine called for changes to improve hospital work environments to make patient care safer and less error-prone [36]—despite this, we still see that poor work environments, and thus nurse burnout, are widespread across the US health system. The COVID-19 pandemic has highlighted how widespread clinician burnout is, but our study and others indicate that poor working conditions contributing to burnout were in place before the pandemic. One recent study that surveyed nurses between December 2019 and February 2020 found that nurses were already overwhelmed with high workloads, missing necessary supplies, and having to deal with inoperable equipment [66]. Notably, half of these nurses reported burnout and a quarter indicated they would leave their job within a year. The additional strain that COVID-19 has placed on healthcare providers makes our findings and their implications for leveraging the work environment as a solution even more relevant.

During hospitalization, every patient receives care from a nurse. Nurses at the bedside are tasked with continuous patient surveillance and medication and treatment administration while serving as the first responder in the event of rapid patient decline. To provide safe, high quality care, nurses need the resources and support to do so, otherwise they become overextended, resulting in burnout. While there have been prior calls to transform work environments to improve the delivery and safety of patient care [36,67], recent efforts by the National Academy of Medicine [68] and other researchers highlight the natural synergy between clinician well-being and better patient outcomes. Specifically, Bodenheimer and Sinsky called for an expansion of the Triple Aim (i.e., improving population health, improving patient experience, reducing costs) [69] to the Quadruple Aim (which adds reducing burnout), arguing that achieving the three aims is unlikely without concentrated efforts to address burnout among healthcare providers [70]. Our results suggest that hospital administrators looking to simultaneously alleviate nurse burnout and improve patient outcomes could achieve both by improving their work environments. The Magnet recognition is an empirically supported approach shown to improve work environments for nurses [71].

### Limitations

This study has limitations, including the use of cross-sectional data, which restricts our ability to draw causal inference. However, a major strength of this study was our use of clinical patient outcomes, as previous research has largely relied on indirect measures like nurse perceived quality and safety. The consistency we observe between our study’s findings and those of previous studies suggests that we should also value the conclusions of studies using indirect measures. We also used a robust patient risk adjustment in addition to controlling for certain hospital features to further isolate the relationship between nurse burnout and mortality, failure to rescue, and length of stay. Another potential limitation is that our measures of burnout and the work environment were created by aggregating the responses of nurses within each hospital to avoid the bias associated with individual responses. To achieve this, we used hospitals with a minimum of 10 nurse respondents. Previous research has shown that having at least 10 nurse respondents per hospital provides an accurate summary of nurse-sensitive hospital features [1,39,40] and our study sample had, on average, 39 nurses per hospital, further minimizing our concern for bias in these measures. Additionally, this is the first study, to the authors’ knowledge, examining the impact of the work environment and Magnet status on the relationship between nurse burnout and patient outcomes, providing greater context for the interpretation of results in addition to highlighting solutions for hospital leaders.

## 5. Conclusions

This study shows how patient lives are dependent on the well-being of the nurses caring for them. In alignment with our first hypothesis, we observed that patients in hospitals with higher levels of nurse burnout had higher odds of mortality and failure to rescue, as well as longer length of stay. While we found that higher nurse burnout was associated with increased risk of these negative patient outcomes, the investigation of our second hypothesis showed that the work environment could be leveraged to minimize this effect. For our final hypothesis, we undertook an analysis of Magnet hospitals and found that they were associated with a reduction in nurse burnout’s effect on mortality and failure to rescue. We suggest that hospital administrators look towards the Magnet model to transform their work environments to minimize burnout and support nurses in providing high quality patient care.

## Figures and Tables

**Table 1:** Distribution of Hospital Characteristics by Average Score of Nurse Burnout.

	All Hospitals (*n* = 523)	Quartile 1 (*n* = 132)	Quartile 2 (*n* = 130)	Quartile 3 (*n* = 131)	Quartile 4 (*n* = 130)	*p ^a^*
**Average Nurse Burnout Score, mean (SD)**						<0.001
	21.0 (3.6)	16.6 (1.9)	19.8 (0.6)	21.9 (0.7)	25.7 (2.1)	
**Nurse Work Environment, *n* (%)**						<0.001
Poor	131 (25.1)	7 (5.3)	14 (10.7)	39 (29.8)	71 (54.2)	
Mixed	262 (50.1)	50 (19.1)	72 (27.5)	83 (31.7)	57 (21.8)	
Good	130 (24.9)	75 (57.7)	44 (33.9)	9 (6.9)	2 (1.5)	
**Magnet Status, *n* (%)**						<0.001
Magnet	83 (15.9)	31 (37.4)	28 (33.7)	15 (18.1)	9 (10.8)	
**Number of Beds, *n* (%)**						0.021
≤100	39 (7.5)	18 (46.2)	5 (12.8)	6 (15.4)	10 (25.6)	
101–250	220 (42.1)	57 (25.9)	49 (22.3)	61 (27.7)	53 (24.1)	
>250	264 (50.5)	57 (21.6)	76 (28.8)	64 (24.2)	67 (25.4)	
**Teaching Status, *n* (%)**						0.046
None	224 (42.8)	68 (30.4)	53 (23.7)	55 (24.6)	48 (21.4)	
Minor	250 (47.8)	54 (21.6)	60 (24.0)	61 (24.4)	75 (30.0)	
Major	49 (9.4)	10 (20.4)	17 (34.7)	15 (30.6)	7 (14.3)	
**Technology Status, *n* (%)**						0.596
High	279 (53.4)	65 (23.3)	74 (26.5)	68 (24.4)	72 (25.8)	
**State, *n* (%)**						0.092
California	207 (39.6)	62 (30.0)	52 (25.1)	52 (25.1)	41 (19.8)	
New Jersey	57 (10.9)	15 (26.3)	19 (33.3)	13 (22.8)	10 (17.5)	
Pennsylvania	110 (21.0)	21 (19.1)	23 (20.9)	27 (24.6)	39 (35.5)	
Florida	149 (28.5)	34 (22.8)	36 (24.2)	39 (26.2)	40 (26.9)	

Abbreviations: SD = standard deviation, *n* = number. Note: Poor environments are hospitals in the bottom 25%, mixed work environments are the middle 50%, and good work environments are the top 25% of hospitals. *p ^a^* values generated from χ2 for categorical and ANOVA for continuous variables. Quartile 1 mean = 16.6, range 8.3–18.6; Quartile 2 mean = 19.8, range 18.6–20.8; Quartile 3 mean = 21.9, range 20.8–23.2; Quartile 4 mean = 25.7, range 23.2–31.9.

**Table 2:** Descriptive Characteristics of Nurse Sample.

Nurse Demographics	All Nurses (*n* = 20,406) No. (%)
Age (years), mean (SD)	48.0 (12.2)
Sex	
Female	18,400 (90.2)
Male	1954 (9.6)
Education	
Diploma/Associates	8108 (39.7)
Baccalaureate	9852 (48.3)
Masters/Doctorate	2376 (11.6)
Years of Experience, mean (SD)	20.2 (13.1)
Position	
Direct Care Staff	15,645 (76.7)
Nurse Manager/Administration	1643 (8.1)
Other Nursing Role	2991 (14.7)

Abbreviations: SD = standard deviation, *n* = number. Note: The minimum number of nurses reporting on demographics was at least 20,279 in all cases.

**Table 3:** Descriptive Characteristics of Patient Sample.

	All Surgical Patients No. (%)
**Patient Demographics**	
Age (years), mean (SD)	62.0 (16.6)
Men	887,507 (45.8)
Race/Ethnicity	
White	1,367,827 (70.5)
Black/African American	170,594 (8.8)
Hispanic	270,792 (14.0)
Asian/Pacific Islander	61,714 (3.2)
Native American	3434 (0.2)
Other	44,363 (2.3)
Transfer status	45,138 (2.3)
**Surgical Group**	
General Surgery	691,867 (35.7)
Orthopedic Surgery	993,636 (51.2)
Vascular Surgery	254,375 (13.1)
**Comorbidities**	
Hypertension	1,099,302 (56.7)
Obesity	326,084 (16.8)
Diabetes without chronic complications	307,979 (15.9)
Chronic pulmonary disease	301,960 (15.6)
Fluid and electrolyte disorders	293,505 (15.1)
Deficiency anemias	260,988 (13.5)
Hypothyroidism	247,901 (12.8)
Depression	202,213 (10.4)
Renal Failure	179,447 (9.3)
Diabetes with chronic complications	130,965 (6.8)
Peripheral vascular disease	127,437 (6.6)
Other neurological disorders	102,772 (5.3)
Congestive heart failure	83,853 (4.3)
Valvular disease	72,481 (3.7)
Liver Disease	69,653 (3.6)
Coagulopathy	68,125 (3.5)
Weight loss	61,047 (3.2)
Rheumatoid arthritis/collagen vas	59,997 (3.1)
Alcohol abuse	59,426 (3.1)
Psychoses	57,581 (3.0)
Metastatic cancer	45,292 (2.3)
Drug abuse	45,302 (2.3)
Paralysis	30,630 (1.6)
Solid tumor without metastasis	25,703 (1.3)
Chronic blood loss anemia	19,499 (1.0)
Pulmonary circulation disease	17,892 (0.9)
Lymphoma	9211 (0.5)
Peptic Ulcer Disease with bleeding	8784 (0.5)
AIDs	3174 (0.2)
**Outcomes**	
30-day in-hospital mortality	15,000 (0.8)
Failure to rescue	12,858 (4.2)
Length of stay (days), mean (SD)	4.3 (5.3)

Abbreviations: SD = standard deviation, *n* = number. Note: (*n* = 1,939,878). Transfer status denotes transferred vs. not transferred.

**Table 4:** Effects of Burnout, Work Environment, and Magnet Status on Patient Outcomes.

Patient Outcomes	Model 1: Unadjusted		Model 2: Hospital Characteristics		Model 3: Patient Characteristics		Model 4: Work Environment		Model 5: Magnet		Model 6: Magnet & Work Environment	
	OR (95% CI)	*p ^a^*	OR (95% CI)	*p ^a^*	OR (95% CI)	*p ^a^*	OR (95% CI)	*p ^a^*	OR (95% CI)	*p ^a^*	OR (95% CI)	*p ^a^*
**30-day in-hospital mortality**												
Burnout	1.06 (1.02, 1.11)	0.003	1.07 (1.02, 1.11)	0.004	1.05 (1.01, 1.10)	0.023	0.97 (0.92, 1.03)	0.310	1.03 (0.98, 1.07)	0.242	0.97 (0.92, 1.02)	0.289
Work Environment	--		--		--		0.86 (0.81, 0.92)	<0.001	--		0.89 (0.83, 0.95)	0.001
Magnet	--		--		--		--		0.82 (0.75, 0.90)	<0.001	0.86 (0.79, 0.94)	0.001
**Failure to Rescue**												
Burnout	1.05 (0.99, 1.10)	0.086	1.06 (1.00, 1.12)	0.043	1.05 (1.00, 1.09)	0.037	0.98 (0.93, 1.04)	0.510	1.03 (0.98, 1.07)	0.230	0.98 (0.93, 1.04)	0.496
Work Environment	--		--		--		0.88 (0.83, 0.95)	<0.001	--		0.91 (0.85, 0.98)	0.009
Magnet	--		--		--		--		0.87 (0.79, 0.95)	0.003	0.90 (0.82, 0.99)	0.038
**30-day length of stay (IRR)**												
Burnout	1.02 (1.00, 1.05)	0.038	1.02 (1.00, 1.04)	0.043	1.02 (1.00, 1.03)	0.013	1.00 (0.98, 1.01)	0.722	1.01 (1.00, 1.03)	0.030	1.00 (0.98, 1.01)	0.722
Work Environment	--		--		--		0.96 (0.94, 0.99)	0.003	--		0.96 (0.94, 0.99)	0.004
Magnet	--		--		--		--		0.99 (0.95, 1.02)	0.464	1.00 (0.97, 1.04)	0.927

Abbreviations: CI = confidence interval, OR = odds ratio, IRR = incidence rate ratio. Note: Burnout represents the average burnout score of nurses in a hospital. The odds ratio reflects a 1 standard deviation change in the average score of burnout. Hospital characteristics include bed size, teaching status, technology status, and state. Patient characteristics include age, sex, race, transfer status, comorbidities, and surgical procedure. Work environment is categorized as poor, mixed, and good. The odds ratio reflects the change in the work environment from poor to mixed or mixed to good.

## Data Availability

The nurse survey data presented in this study are not publicly available due a certificate of confidentiality for the respondents of the nurse survey and data use agreements for patient data.
